# Nitrogen Fertilization Influences the Quantity, Composition, and Tissue Association of Foliar Phenolics in Strawberries

**DOI:** 10.3389/fpls.2021.613839

**Published:** 2021-04-20

**Authors:** Ashwini Sushil Narvekar, Nishanth Tharayil

**Affiliations:** Department of Plant and Environmental Sciences, Clemson University, Clemson, SC, United States

**Keywords:** nitrogen, polyphenols, flavonoids, tannins, metabolomics

## Abstract

Unlike quantitative changes, the compositional changes of plant phenolics and changes in their tissue association as influenced by the nutrient supply are less well understood. We evaluated the quantity, composition, and tissue association of phenolics in leaves of two *Fragaria ananassa* cultivars in response to different levels of nitrogen (N) fertilization using global metabolomic approaches. Influence of N supply on phenolic content in both cultivars was similar, but the magnitude of this response was compound specific. Ellagitannins, the most abundant class of phenolic oligomers, were less responsive to the applied N treatments, whereas proanthocyanidins, the less abundant class of phenolic oligomers, exhibited higher fold change. Within mono-phenolics, the hydroxycinnamates were more abundant but showed lower fold change than the hydroxybenzoates. Among flavonoids, the hydroxylated flavonols showed higher abundances than the flavones, with a preferential accumulation of dihydroxylated flavonol at lower N levels. Furthermore, glycosylated flavonols were higher than the acylated forms. The extractable fraction of phenolics was more influenced by the N treatment than the fiber-bound fraction. The extensive compositional modification of phenolics and a greater response of non-bound fractions in response to N rates highlight the potential to use precise management of N supply as an effective strategy to enhance the bioactive compounds in crops.

## Introduction

Secondary metabolites play a myriad of essential roles in plant–organismal and plant–environmental interactions, including functioning as signaling molecules, structural polymers, attractants and deterrents, regulators of phytohormones, and stress mitigators (Dixon et al., 2005). Secondary metabolite production in plants is influenced by the plant genotype, the environment, and their interaction; hence, the composition of secondary metabolites in plants is highly diverse (Wurtzel and Kutchan, 2016). This compositional diversity confers a broader array of biological functions to the secondary metabolites. The molecular identity also influences the association of metabolites with different cellular compartments, which in turn guides the overall function of these compounds in plants. Although the changes in the overall quantity of secondary metabolites in relation to environmental stressors have been widely studied, we lack an in-depth understanding of the stress-induced changes in the composition and tissue association of secondary metabolites. This knowledge could be critical to forecasting the biological role of these compounds in facilitating overall plant fitness.

**FIGURE 1:**
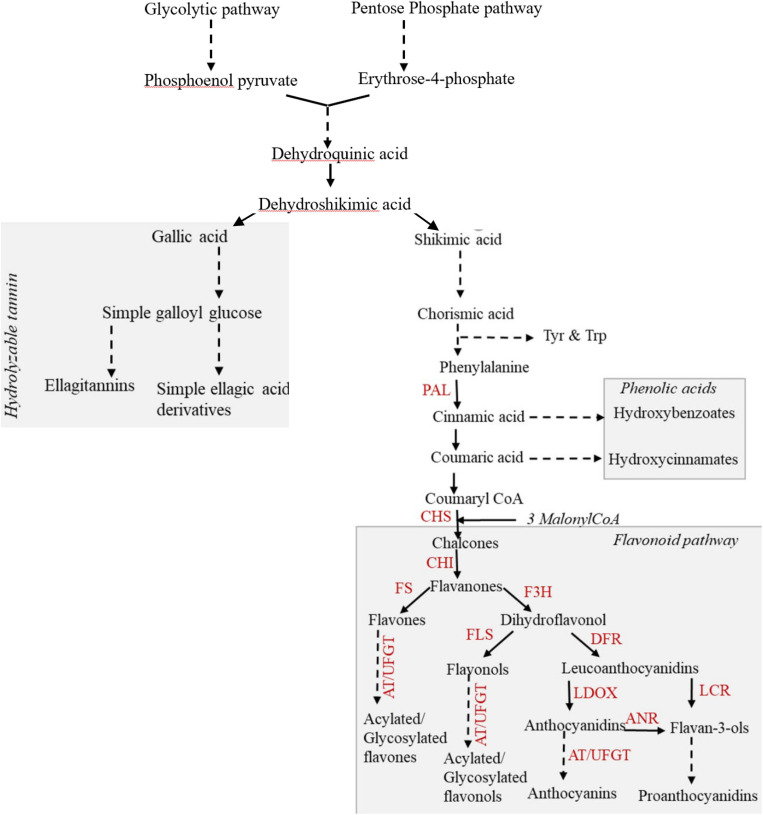
Scheme of the biosynthesis of phenolics in plants focusing on the flavonoid classes and the potential trade-off between the HTs and flavonoids. Discontinuous lines indicate multiple steps that are not all included in the figure. Phenolic acids and flavonoids together constitute the phenylpropanoids. The key enzymes that regulate this biosynthesis are indicated: PAL, phenylalanine ammonia lyase; CHS, chalcone synthase; CHI, chalcone isomerase; FS, flavone synthase; F3H, flavanone3-hydroxylase; FLS, flavonol synthase; DFR, dihydroflavonol 4-reductase; LDOX, leucoanthocyanidin dioxygenase; LCR, leucoanthocyanidin reductase; ANR, anthocyanidin reductase; AT, acyltransferase; UFGT, UDP flavonoid glucosyltransferase.

Phenolic compounds (phenolics) constitute one key class of carbon-based secondary metabolites that are of ecological and nutritive importance. Phenolic metabolites are biosynthesized from the derivatives of the shikimate pathway and are broadly categorized as phenolic acids, flavonoids, and tannins (Vogt, 2010). The phenolic acids and flavonoids together constitute the phenylpropanoids, whereas the tannins encompass two classes, namely, hydrolyzable tannins (HTs) and proanthocyanidins (condensed tannins; Suseela and Tharayil, 2018). The shikimate pathway intermediate *viz.* dehydroshikimate is utilized in the biosynthesis of gallic acid, the monomer unit for HT (Figure 1). Gallic acid monomers ester-link to glucose form simple galloyl glucoses, which further condense to form gallotannins (GTs) or ellagitannins (ETs; Salminen and Maarit, 2011; Top et al., 2017). The terminal product of the shikimate pathway, chorismic acid, is the precursor of phenylalanine, the precursor of phenylpropanoids. Deamination of phenylalanine generates phenolic acids, hydroxybenzoates (C_6_–C_1_), and hydroxycinnamates (C_6_–C_3_), which are then utilized for the synthesis of diverse salicinoids and flavonoid compounds. The flavonoids are further categorized into six broad groups based on the chemical structure derived from the enzyme-catalyzed modification of the basic flavan scaffold (C_6_–C_3_–C_6_) (Winkel-Shirley, 2002; Vogt, 2010): flavanones, flavones, flavonols, flavan-3-ols, anthocyanins, and proanthocyanidins (Ferreyra et al., 2012; Alseekh et al., 2020). Diversity within the polymeric phenolic compounds that include the proanthocyanidins and HTs is attributed to the variation in the type, number, and linkage between the monomers (Xie and Dixon, 2005; Salminen and Maarit, 2011). Although previous studies have tracked the changes in content of phenolic compounds as a function of environmental stress, the environment-induced changes in the composition and localization of these metabolites remain less understood.

The stress mitigation efficiency of phenolic compounds is greatly influenced by their molecular structure (Heim et al., 2002; Alseekh et al., 2020). For example, compared to mono-phenolics, flavonoids have a higher antioxidant capacity due to the enhanced electron-donating properties of the catechol group in the B-ring. Since the radical scavenging activity of flavonoids is affected by the hydrogen donation of its phenolic hydroxyl group, the antioxidant capacity increases with the number of hydroxyl groups in the B-ring (Heim et al., 2002). Along with the number of hydroxyl groups, their position on the B-ring also contributes to the antioxidant potential. For example, compared to an ortho-diphenolic structure of quercitin, the meta-arrangement of dihydroxyl groups reduces the antioxidant potential of the morin by half. Within the flavonoids, the flavonols and flavones have conjugations between the A- and B-ring and thus a more efficient electron delocalization across the molecule. Hence, they have a higher antioxidant capacity than flavanones, which have a saturated C-ring. Furthermore, saturation of the C-ring and the hydroxylation of the A- and B-ring interact to modify the overall antioxidant potential of flavonoids (Heim et al., 2002). Similarly, the glycosylation of flavonoids reduces their antioxidant potential, as it affects the delocalization of electrons but this modification facilitates their transport within plants (Kähkönen and Heinonen, 2003; Cyboran-Mikołajczyk et al., 2018).

Along with the above compositional changes, under stress, the structural changes determine the association (localization) of the phenolics with cell wall. Phenolics exist either as extractable (soluble) forms present mainly in vacuoles or as non-extractable forms that are attached to cell wall proteins and carbohydrates (fiber-bound). The localization of phenolics significantly influences their function, with polyphenols conjugated to cell walls exhibiting a higher photoprotection efficiency than those sequestered in cell vacuoles (Clarke and Robinson, 2008). For example, the flavonoid molecules sequestered in vacuoles serve as antioxidants by quenching reactive oxygen species (ROS), and the same molecule when bound to the cell wall serves as a photoprotectant by absorbing UV-A radiation. The localization of phenolics also affects their transport between sources (e.g., leaf) and sinks (e.g., fruits) (Cadot et al., 2011; Allegro et al., 2016; Shahidi and Yeo, 2016) and thus can influence the nutritive quality of produce. Despite the influence that the chemical composition and localization have on their biological activity, phenolic compounds have less frequently been subjected to finer-level compositional studies.

Nitrogen (N) is a key macronutrient, and its availability dictates the partitioning and transportation of photosynthetic assimilates between the primary and secondary metabolism in plants (Bot et al., 2009; Tegeder and Masclaux-Daubresse, 2018; Fernie et al., 2020). The major theories proposed to explain the variation in the biosynthesis of secondary metabolites in response to N availability include the carbon nutrient balance theory (Coley et al., 1985), the growth–differentiation balance theory (Herms and Mattson, 1992), and the protein competition model (Jones and Hartley, 1999). According to these theories, under N limitation, plants direct the accumulation of carbon-based assimilates toward the synthesis of secondary compounds. Along these lines, many crop species have shown increased phenolic content under N limitation (Fritz et al., 2006; Larbat et al., 2012; Becker et al., 2015). However, the compositional changes of phenolics as influenced by plant ontogeny and the amount and form of N fertilization are less known. Foliar phytochemistry influences plant vigor through stress mitigation (Bobeica et al., 2015), susceptibility to pest and pathogens through deterrence (Kim et al., 2016), and nutritive quality of the yield through source–sink interactions (Fernie et al., 2020). Phytochemicals produced in the leaves are transported to fruits (Petrussa et al., 2013; Gutierrez et al., 2017), making the foliar phytochemistry a key determinant of the content and quality of phytochemicals of the fruits. This transport of the phytochemicals out of leaves is primarily determined by the association of the compounds with the cellular matrix (Top et al., 2017). Compared to phytochemicals that are ester/ether-linked to cell walls (cell wall-bound), compounds that are sequestered in vacuoles are readily transported across the cells and organs. Thus, the association of phytochemicals within the foliage could influence the chemical composition of the fruits. However, little is known about the influence of N limitation on the reprogramming of the tissue association of phenolics.

The metabolome, which is the end product of gene expression, could effectively capture the physiology of stress mitigation in plants (Alseekh and Fernie, 2018). Thus, metabolomics could be an ideal tool to understand plant ecophysiology, which could pave the way for developing new biotechnological solutions benefiting both plants and the environment (Alseekh et al., 2018). The main objectives of this study were to evaluate how different levels of N fertilization affect the content, composition, and association of different classes of foliar phenolics. We hypothesized that (i) the significant reprogramming of phenylpropanoid pathway under N stress would not only result in quantitative changes in the total phenolic content but also impart changes in their composition, whereby the proportional abundance of different classes of phenolics and the glycosylation within a class would be influenced by N; (ii) the altered N supply would lead to the differential association of the biosynthesized phenolics with the tissue fraction (soluble vs. bound), and the proportion of soluble-to-bound phenolics would be higher with increased N limitation; (iii) above changes in composition and localization of phenolic compounds could exhibit a non-linear relationship with N availability.

## Materials and Methods

### Plant Source

Strawberry plants (*Fragaria ananassa*) cv. Camarosa (June-bearing) and cv. Albion (day-neutral) were procured as plugs (3–5 leaves) from Lassen Canyon Nursery, Inc., Redding, CA, United States. The plants were grown at Clemson University, SC, United States, in a greenhouse in 7-L plastic pots with ∼2 kg potting mixture, which had a base N level of 7.5 μg/g (150 mg N/pot). The treatment application was initiated 2 months after the plugs were transplanted to the pots. Four levels of N treatment were evaluated in the study, consisting of the control (0 N), 8 mM N, 16 mM N, and 30 mM N. Nitrogen fertilizer was supplied in the form of ammonium nitrate, and the treatment solution was applied as 200 ml/pot at an interval of 10 days. This fertilizer dosage corresponds to an N addition of 0, 43, 86, and 160 mg/pot every 10 days. The treatment time and rate of application were selected based on the preliminary experiments on N addition, with the 30-mM N treatment being closer to the recommend rate of N application for strawberries in the Southeastern United States under annual plasticulture production systems (Rysin et al., 2015). The treatment consisted of a completely randomized design with five biological replicates. Twelve weeks after treatment, mature actively growing young leaves were harvested from the third whorl of each plant and immediately frozen using dry ice. The leaves of similar leaf area were selected across the treatment, and at the time of harvest, none of the plants, including the control treatment, exhibited visible chlorosis. The tissues were ground into a fine powder with dry ice and immediately transferred to −80°C for storage until further analysis.

### Estimation of Primary Metabolites by Gas Chromatography–Mass Spectrometry

Polar metabolites were extracted from the ground tissue as per Suseela et al. (2015), with slight modification. Briefly, 300 mg of powdered plant material was extracted with 3 ml of methanol using a bead-beater homogenizer (ceramic beads, 6,000 rpm, 2 min) and sonication (25% amplitude, 1 min) and centrifuged at 1,500 × *g*. The samples were kept at 4°C during the processing. One milliliter of supernatant was transferred to a glass tube, and the extract was further partitioned to polar and non-polar phase by successive addition and mixing 1 ml of ice-cold CHCl_3_ and water. The tubes were centrifuged at 1,500 × *g* to complete the phase separation between the lower CHCl_3_ phase and the top aqueous methanol phase. Forty microliters of the aqueous methanolic supernatant was transferred to silanized glass inserts with 20 μl of 200 μg/ml ribitol (internal standard) and 500 μg/ml d_27_-myristic acid (retention time lock). The samples and standards were completely dried in a vacuum evaporator at room temperature. Twenty microliters of a 40-mg/ml solution of methoxylamine hydrochloride in pyridine was added to the glass insets and incubated at 40°C for 60 min with intermittent mixing. The second derivatization consisted of the addition of 90 μl of N-methyl-N-(trimethylsilyl) trifluoroacetamide (MSTFA) with 1% trimethylchlorosilane (TMCS) and incubating the samples for 30 min at 55°C. The derivatized samples were analyzed on a gas chromatograph coupled to a mass spectrometer (GC-MS; Agilent 7980, Agilent Technologies, Santa Clara, CA, United States). Compounds were separated on a DB-5 column. The GC-MS instrument settings were as per Maroli et al. (2015). Mass spectra were processed with an automatic mass spectral deconvolution and identification system (AMDIS v2.71, NIST, Gaithersburg, MD, United States) with a minimum match factor of 70. The identification of the compounds was based on their retention time index and mass and fragmentation pattern comparison with the Fiehn library (G1676AA; Agilent Technologies, Wilmington, DE, United States; Suseela et al., 2015). The peak intensities were normalized to ribitol.

### Estimation of Proanthocyanidins by Acid Butanol Assay

The total proanthocyanidins in the leaf sample were estimated by acid butanol assay modified from Top et al. (2017). The aqueous methanol phase was dried at room temperature in a vacuum evaporator, followed by the addition of 1 ml of acid butanol reagent and incubation at 95°C for 60 min. A spectrophotometric measurement of the color that developed was taken at 550 nm, and the amount of proanthocyanidins was calculated using a standard curve derived from the cyanidin standard.

### Estimation of Extractable Phenolics by Liquid Chromatography–Mass Spectrometry

Briefly, 600 mg of ground leaf tissue was extracted with 4 ml of methanol and homogenized at 4°C as described above and centrifuged 3,000 rpm. The sediment was then sequentially extracted twice with methanol and thrice with 80% aqueous acetone to completely extract the vacuolar metabolites. The supernatant from all six extractions was pooled and was used for the determination of soluble (extractable) phenolics. Isotope-labeled resveratrol (^13^C_6_; 0.5 ng/μl) was added as the internal standard. The samples were analyzed on an Ultimate 3000 HPLC (ultrahigh-pressure liquid chromatography) coupled to an Orbitrap Fusion Tribrid mass spectrometer equipped with electrospray ionization (ESI; Thermo Scientific, Waltham, MA, United States) as per Bowers et al. (2018). Separation of the metabolites was carried out using an HSS T3 column (Waters Corp., Milford, MA, United States; 150 mm × 2.1 mm, 1.8 μm) at 30°C. The following gradient program utilizing water with 0.1% formic acid as mobile phase A and acetonitrile as mobile phase B was employed: 0 min, 5% B; 2 min, 5% B; 24 min, 60% B; 27 min, 90% B; followed by a 3-min washing step at 90% B and a subsequent re-equilibration for 6 min at 5% B. The flow rate was set to 0.22 ml/min, and the injection volume was set as 2 μl. The mass spectrometer was operated as per Bowers et al. (2018) in negative ionization mode with a data-dependent MS^2^ HCD-CID method for compound confirmation. The interface conditions were as follows: emitter voltage, −2,600 V; vaporizer temperature, 325°C; ion transfer tube, 325°C; sheath gas, 55 (arb); aux gas, 10 (arb); and sweep gas, 1 (arb).

### Estimation of Bound Phenolics by Liquid Chromatography–Mass Spectrometry

The bound (non-extractable) phenolics were determined on the residue left after the sequential extraction with methanol and acetone. The residue was dried at 60°C and acid hydrolyzed using 2 M methanolic HCl with 0.04% ascorbic acid at 85°C for 4 h. The supernatant after centrifugation was used for the determination of the bound phenolics (Top et al., 2017). The samples were analyzed using an Orbitrap Fusion Tribrid mass spectrometer as described above.

### Determination of Antioxidant Capacity

The extract from the extractable phenolics for liquid chromatography–mass spectrometry (LC-MS) analysis was used for the determination of the antioxidant capacity of the strawberry leaves from the different N treatments using a DPPH assay (Xie and Schaich, 2014). Briefly, 100 μl of the extract was added to 900 μl of freshly prepared 1 mM methanolic DPPH reagent and incubated at room temperature in the dark. After 30 min, the absorbance was measured at 515 nm against the reagent blank. The percentage of DPPH radical scavenging was calculated as given in Eq. 1, where *A*_0_ is the absorbance of the reagent without the sample, and *A* is the absorbance of the reaction mixture with the sample solution.

(1) $$\text{Inhibition}\%=\left\{\left(A_{0}-A\right)/A_{0}\right\}\times 100$$

### Data Analysis and Statistics

Statistical significance was determined by one-way ANOVA followed by Tukey’s multiple comparisons for normally distributed data and by Kruskal–Wallis ANOVA for non-parametric data using JMP Pro13.1.0 (SAS Institute Inc., Cary, NC, United States) with a statistical significance level of *P* < 0.05. To evaluate the effect of the applied N treatments on the overall foliar metabolite profile, the metabolomics data were subjected to multivariate analysis using Metaboanalyst 4.0 (Chong et al., 2018). The metabolomic data of the two cultivars were autoscaled or log-transformed to satisfy the assumptions of normality. The metabolites were further analyzed with partial least squares discriminant analysis (PLS-DA) along with built-in cross-validation and permutation testing. The tentative identification of the phenolic metabolites was based on the literature reporting the accurate masses of the precursor ion and fragment ions using Xcalibur 4.1 (Thermo Fisher Scientific, MA, United States). The data were processed by the node-based workflow of the Compound Discoverer 2.2 software (Thermo Fisher Scientific, MA, United States). The ^13^C resveratrol normalized peak area was utilized to evaluate the phenolic metabolites quantitatively. Graphs were plotted using GraphPad Prism 7.05 (GraphPad software, La Jolla, CA, United States).

## Results

### Variation Among the Primary Metabolites as a Response to the Nitrogen Supply

**FIGURE 2:**
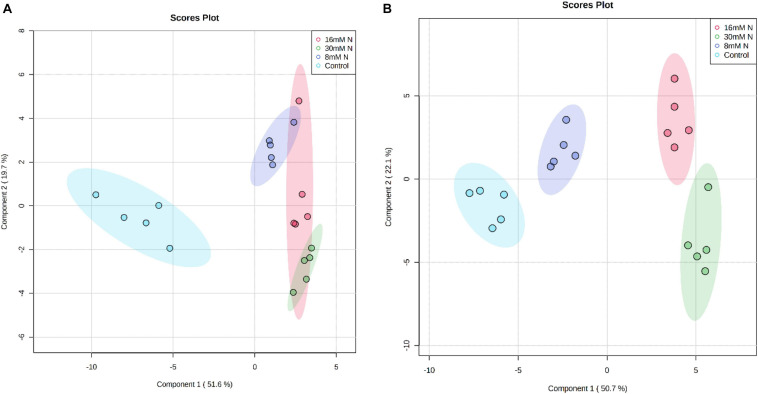
Partial least squares discriminant analysis (PLS-DA) score plot of the first two principal components of the analysis of polar primary metabolites of strawberry cv. Albion **(A)** and cv. Camarosa **(B)** exposed to four levels of nitrogen (Green: 30 mM N, Red: 16 mM N, Blue: 8 mM N, and Turquoise: Control). The permutation cross-validation of the PLS-DA model had *P* = 0.0005 over 2,000 iterations. The ellipse represents a 95% confidence interval, and data points represent biological replicates (*n* = 5).

The composition of primary metabolites in strawberry leaves, which included sugars, sugar alcohols, organic acids, and amino acids, was quantified using a GC-MS-based metabolomic approach. The analysis identified 40 and 49 metabolites from the leaf extract of cv. Albion and cv. Camarosa, respectively. PLS-DA of these metabolites along the first two-component axis (Figure 2) explained *ca*. 73–78% of the total variation in the data. The PLS-DA analysis showed good model fitness with *R*^2^ and *Q*^2^ value of >0.6. The robustness of the class discrimination was verified through permutation testing of separation distance based on the ratio of the between-group sum of squares and the within-group sum of squares and had *P* < 0.05 over 2,000 iterations. The metabolites with variable importance in projection (VIP) scores >1 are considered relevant for influencing the grouping (Supplementary Figure 1). In both cultivars, the component axis 1 separated control treatment from the other N treatments, and all other N treatments grouped into distinct clusters along the component axis 2. Although the metabolites possessing the highest discrimination potential varied between the two cultivars, they predominantly constituted tagatose, trehalose, citric acid, fumaric acid, and galactitol. All metabolites that discriminated between the treatments did not exhibit a linear response to the level of N additions.

**FIGURE 3:**
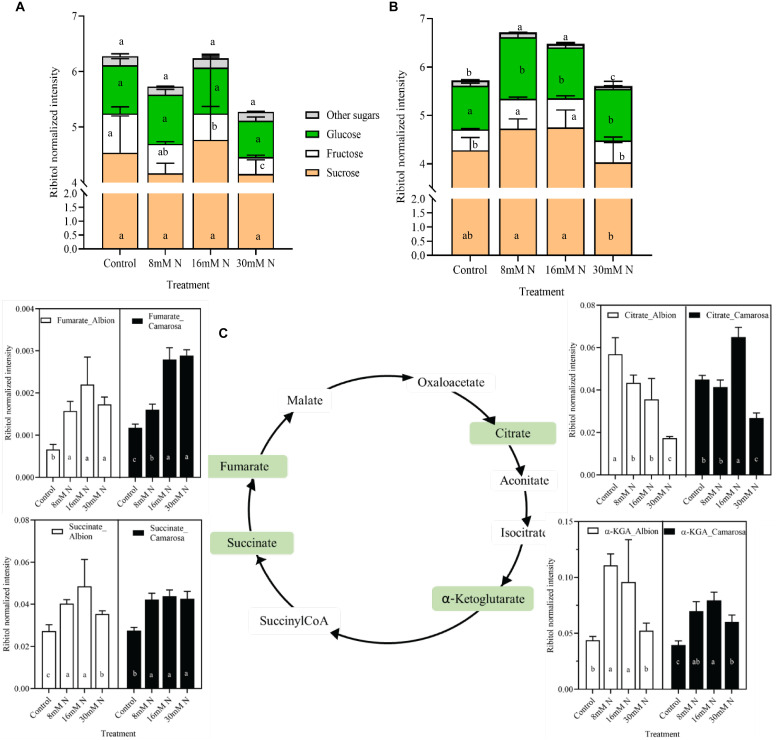
Abundance of sugars in cv. Albion **(A)** and cv. Camarosa **(B)** and intermediaries of tricarboxylic acid (TCA) cycle **(C)** of cv. Albion (open bars) and cv. Camarosa (closed bars), as influenced by the four levels of N treatments. The data represent the mean ± SD (*n* = 5) ribitol normalized abundance of the respective samples and were tested by one-way ANOVA with Tukey’s honestly significant difference (HSD). Bars with the same letters are not significantly different at the 95% confidence interval.

The relative abundances of the total non-structural sugars (Figures 3A,B) exhibited the least variation between N-replete (30 mM N) and -deficient (control) treatment for both cultivars. Sucrose was the most abundant sugar in both cultivars, contributing to *ca*. 69–80% of the total sugar and did not vary with N treatment. Other soluble sugars like glucose, trehalose, and tagatose exhibited non-linear responses (Supplementary Figure 1) with accumulation at N-limited treatment for the two cultivars. The tricarboxylic acid (TCA) cycle intermediates showed differential response across the treatments in the two cultivars (Figure 3C). Dehydroascorbate and quinate constituted the non-TCA organic acids and exhibited much lower percentage variation across the N treatments.

### Variation in the Content and Composition of Polyphenols in Response to Varied Nitrogen Supply

**FIGURE 4:**
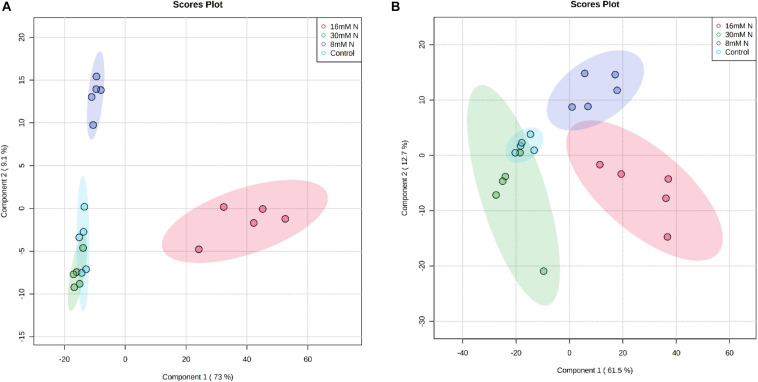
Partial least squares discriminant analysis (PLS-DA) score plot of the first two principal components of the analysis of 789 secondary metabolites of strawberry cv. Albion **(A)** and cv. Camarosa **(B)** exposed to four levels of nitrogen (Green: 30 mM N, Red: 16 mM N, Blue: 8 mM N, and Turquoise: Control). The permutation cross-validation of the PLS-DA model had *P* = 0.0005 over 2,000 iterations. The ellipse represents a 95% confidence interval, and data points represent biological replicates (*n* = 5).

After accounting for multiple adducts, potential source fragmentation, UHPLC-ESI-Orbitrap-MS data analysis of the strawberry leaf tissue using Compound Discoverer 2.2, positively annotated 789 potential compounds across all samples. Among them, 738 and 680 compounds in cv. Albion and cv. Camarosa, respectively, were classified as significant based on the log fold change (2), *P* value, and false discovery rate (<0.05). Score plot from PLS-DA of the 789 metabolites plotted for two discriminatory components explained *ca*. 74–83% of the total variation and separated the different N treatments for both cultivars (Figure 4). The PLS-DA model showed a good model fit with *R*^2^ and *Q*^2^ value >0.6 and *P* value <0.01 based on the permutation testing. The tentative molecular identity of 149 metabolites (Supplementary Table 1) was established based on manual curation based on their accurate molecular masses (<5 ppm), MS^2^ fragmentation pattern from the literature, and online mass spectral libraries (mzCloud, Massbank). The identified phenolic compounds belonged to non-anthocyanin categories of phenolics, which included simple galloyl glucoses, ellagic acid derivatives, ETs, hydroxybenzoates, hydroxycinnamates, flavanones, flavones, flavonols, flavan-3-ols, and proanthocyanidins. Compounds having the same molecular masses (mass error <2 ppm of theoretical mass) but having different retention times within the same sample (>0.5 min) were treated as a unique compound. The cumulative peak area of the identified metabolites constituted *ca.* 54% of the total peak area of the 789 compounds. The PLS-DA of the identified 149 metabolites also differentiated the N treatments along the principal component axis 1 that explained >61% variation, with the control treatments significantly different from the N-addition treatments (Supplementary Figure 2).

**FIGURE 5:**
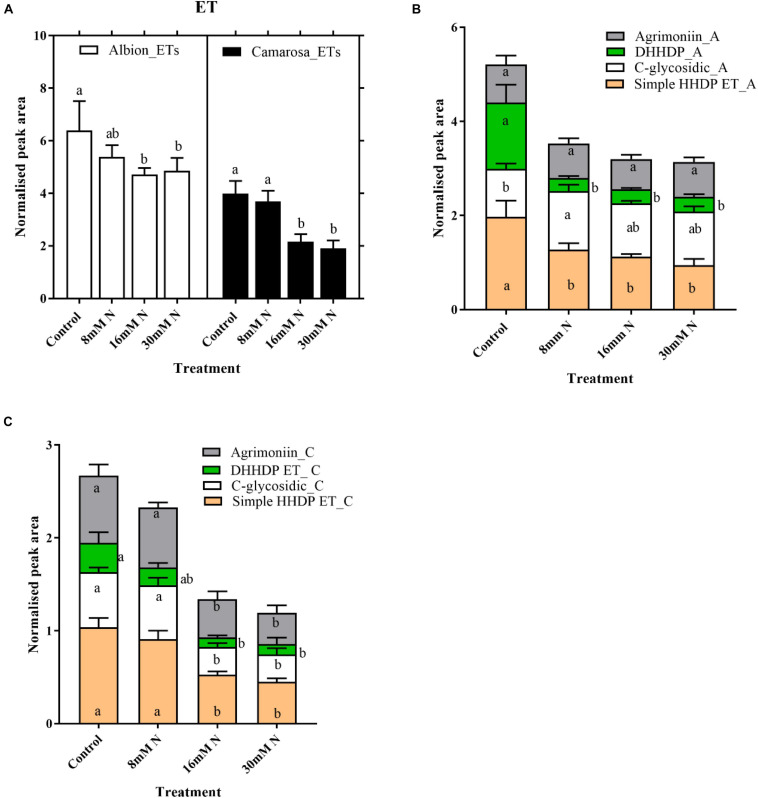
Abundances of total ellagitannin **(A)** and different subgroups of ellagitannins in *Fragaria ananassa* cv. Albion **(B)** and cv. Camarosa **(C)** exposed to four N treatments. The data represent the mean ± SD (*n* = 5) ^13^C resveratrol (1 ng) normalized abundance and were tested by one-way ANOVA with Tukey’s honestly significant difference (HSD). Bars with the same letters are not significantly different at the 95% confidence interval.

**FIGURE 6:**
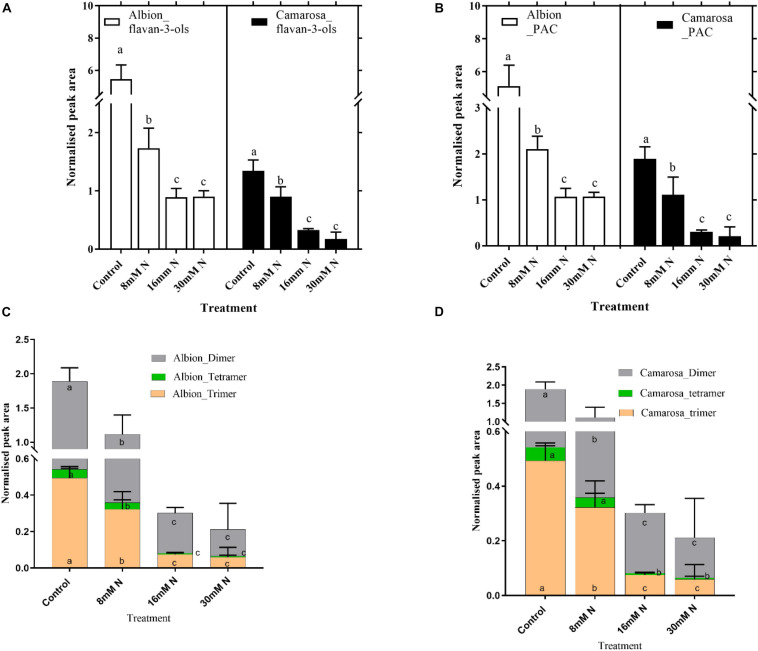
Abundances of flavan-3-ols **(A)** and proanthocyanidins **(B)** and different subgroups of proanthocyanidins in *Fragaria ananassa* cv. Albion **(C)**, and cv. Camarosa **(D)** exposed to four N treatments. The data represent the mean ± SD (*n* = 5) ^13^C resveratrol (1 ng) normalized abundance and were tested by one-way ANOVA with Tukey’s honestly significant difference (HSD). Bars with the same letters are not significantly different at the 95% confidence interval.

Ellagitannins were the most abundant compound class in the leaf metabolites, comprising 48 out of the 149 tentatively identified phenolic compounds and accounts for 23% of the total peak area of the 789 metabolites. For both cultivars, the total ET content increased (*P* = 0.01) with a decrease in N application (Figure 5A). For both cultivars, the content of hexahydroxydiphenoyl (HHDP) ET and dehydro-HHDP esters (geraniin) doubled under lower N treatments (control and 8 mM N) as against N-replete treatment (Figures 5B,C). Galloyl glucoses and tetragalloyl glucoses, the precursors of ETs, were higher in control treatment for both cultivars (Supplementary Figure 3A). Simple ellagic acid derivatives also showed *ca.* 1.5-fold increase (*P* < 0.002) in control treatment as compared to N-replete treatment for both cultivars (Supplementary Figure 3B). Catechin was the most abundant metabolite in both cultivars, accounting for *ca.* 7% of the total peak area of the 789 metabolites. Oligomeric proanthocyanidins and its precursor, flavan-3-ols, increased with a decrease in N availability in both cultivars (Figures 6A,B). In both cultivars, the total flavan-3-ols and oligomeric proanthocyanidins increased linearly with an increase in N addition. The dimers constituted 68–71% of the total proanthocyanidins identified in both cultivars; however, in cv. Camarosa, the trimer and tetramers showed a higher fold increase under N limitation as compared to the dimers (Figures 6C,D). In general, the hydroxycinnamates were 4- to 10-fold higher than hydroxybenzoates. In both cultivars, the hydroxybenzoates increased with decrease in N supply (Supplementary Figure 4).

**FIGURE 7:**
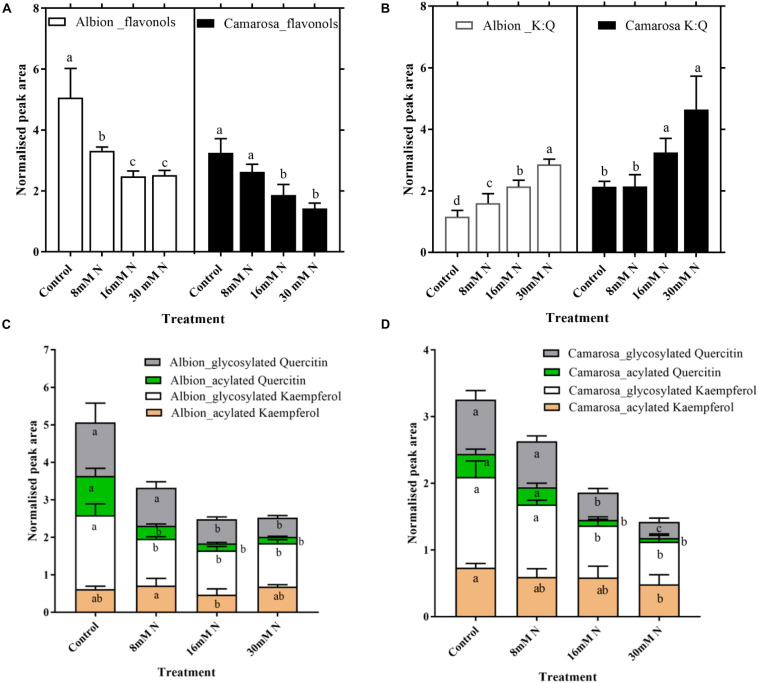
Abundances of flavonols **(A)** and kaempferol:quercetin ratio **(B)** and different structural modifications of kaempferol and quercetin in *Fragaria ananassa* cv. Albion **(C)** and cv. Camarosa **(D)** exposed to four N treatments. The data represent the mean ± SD (*n* = 5) ^13^C resveratrol (1 ng) normalized abundance and were tested by one-way ANOVA with Tukey’s honestly significant difference (HSD). Bars with the same letters are not significantly different at the 95% confidence interval.

Naringenin and eriodictyol glucoside that constituted the flavanone class that is derived from the chalcones showed *ca*. 60% increase (*P* < 0.05) in control as compared to N-replete treatment in both cultivars (Supplementary Figure 4). Flavonols represented the second abundant class of phenolic compounds in strawberry leaves. The flavonol content increased (*P* < 0.005) in both cultivars, with a decrease in N concentration from 16 mM N to control (Figure 7A). Free aglycones of flavonoids in the soluble fraction were not detected, and glycosides of quercetin and kaempferol were the two key flavonols identified in both cultivars. The kaempferol:quercetin ratio decreased by more than half under N-replete treatment in both cultivars at lower N (control and 8 mM) (Figure 7B). About 61–80% of the total tentatively identified flavonoids were glycosylated and 26–38% acylated (Figures 7C,D). Hexoses and pentoses were the only sugars identified to be associated with flavonols along with glucuronide. Only coumaroyl hexoside was an acylated flavonol form identified in both cultivars and constituted about 30% of the total identified flavonols. Coumaroyl quercetin increased (*P* < 0.02) by >5-fold in control treatment of both cultivars as compared to 30 mM N treatment, whereas coumaroyl kaempferol hexoside showed non-significant variation across the N treatments.

### Variation in the Localization of the Polyphenols in Response to Varied Nitrogen Supply

**FIGURE 8:**
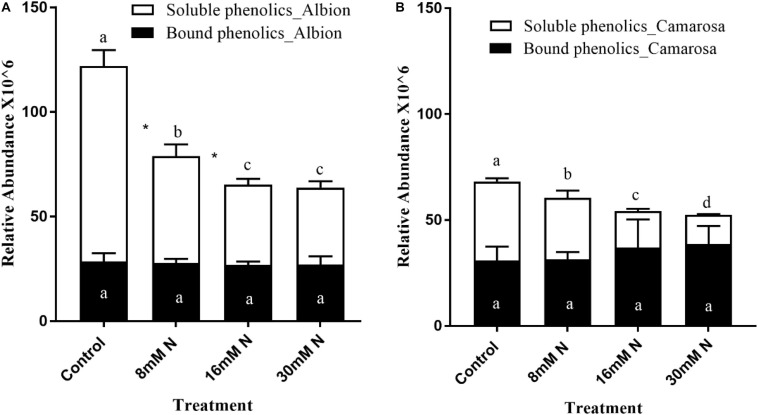
Proportion of soluble vs. fiber-bound phenolics in *Fragaria ananassa* cv. Albion **(A)** and cv. Camarosa **(B)** exposed to four N treatments. The data represent the mean ± SD (*n* = 5). Bars with the same letters are not significantly different at the 95% confidence interval. “*” Denotes statistical significance (*P* < 0.05) between bound and soluble phenolics with a treatment.

Based on accurate mass and fragmentation patterns of phenolics from literature, 17 phenolic aglycone metabolites (Supplementary Table 1) could be tentatively identified from the acid hydrolysis of residue. Total bound phenolics did not show any significant variation to the applied N treatments. Thus, change in the soluble phenolics was the major contributor to the observed change in the proportion of bound to soluble phenolics. The total soluble phenolics were >1.5-fold higher under N limitation compared to N-replete in both cultivars (Figure 8).

Derivatives of protocatechuic acid, coumaric acid, gallic acid, caffeic acid, and isoferulic acid were used for computing the variation between bound and soluble simple phenolic acids. In cv. Albion, bound and soluble simple phenolic acids increased by >50% (*P* < 0.01) in control treatment as compared to N-replete treatments (Supplementary Figure 5A). In cv. Camarosa, the bound-to-soluble ratio decreased by half with a 1.2-fold increase (*P* < 0.001) in the soluble phenolic acids in the control treatment (Supplementary Figure 5B). For both cultivars, the soluble flavonols showed >50% increase (*P* < 0.001) with a decrease in N (Supplementary Figures 5C,D). The sum of ellagic and gallic acid content, the hydrolysis product of bound HTs, did not show any variation to the applied N treatment, and total soluble HT increased by 35–47% and *ca*. 56–91% in cv. Albion and cv. Camarosa, respectively, with the decrease in N from 16 mM N to control (Supplementary Figures 6A,B). In cv. Albion, the total proanthocyanidin content was higher [2.2 mg cyanidin equivalent/g tissue fresh weight (FW)] in N-deficient plants as compared to cv. Camarosa (1.1 mg cyanidin equivalent/g tissue FW). In cv. Albion, 45% of the total proanthocyanidins were in an extractable form in the control treatment, which dropped (*P* > 0.05) to 33% under N-replete levels (30 mM N) (Supplementary Figure 6C). However, in cv. Camarosa, under N limitation, 70% of the total proanthocyanidins were in extractable form, which significantly dropped to 35% under N-replete levels (30 mM N) (Supplementary Figure 6D). The N-deficient plants showed approximately 90 and 88% radical scavenging of 0.1 mM DPPH solution for cv. Camarosa and cv. Albion, respectively (Supplementary Figure 10), whereas the radical scavenging activity decreased to 48 and 59% for cv. Camarosa and cv. Albion, respectively, under N-replete conditions.

## Discussion

### Variation in the Content of Primary Metabolites in Response to the Nitrogen Supply

By regulating the C/N balance, N determines the feedback mechanisms of the biochemical reactions (Paul and Pellny, 2003). As observed on this study, higher N can drive higher tissue carbohydrate content, similar to the positive curvilinear relationship between the soluble sugars and N levels reported in potato leaves, grapes, and corn (Chen and Cheng, 2003; Jin et al., 2015; Braun et al., 2016). This response is thought to stem from a plant strategy of maintaining a balance between photochemistry and physiology, where the production of sugars acts as a non-radiative energy dissipation mechanism (Reyes et al., 2016). In addition to sucrose, which was the most abundant sugar in leaves, other soluble sugars, and sugar alcohols exhibited a much lower and non-linear response across the N treatments (Supplementary Figure 1). These metabolites are known for their role as osmoprotectants, scavengers of ROS, and signaling molecules and are reported to be upregulated under cold, heat, and drought stress (Nishizawa et al., 2008; Koehler et al., 2015). Organic acids derived from the anaplerotic TCA cycle act as transient carbon substrates for amino acid synthesis and as regulators of redox and energy levels (Igamberdiev and Eprintsev, 2016). In the present study, the non-linear variations of the organic acids (Figure 3C) point toward the multifaceted role of the TCA cycle in generating reducing equivalents and α-ketoglutarate for efficient N assimilation (Igamberdiev and Eprintsev, 2016). The abundance of α-ketoglutarate and glutamate under reduced N conditions is in accordance with previous reports in maize and barley (Amiour et al., 2012; Quan et al., 2016).

### Variation in the Content of Phenolic Classes in Response to Varied Nitrogen Supply

The present study captures the changes in the content of phenolics in strawberry leaves under different N levels; however, the magnitude of this change varied among the phenolic classes. The shikimate pathway, the entry point of carbohydrates for the biosynthesis of phenylpropanoids and HTs, represents the first branch point of phenolic biosynthesis. ETs are the major subclass of HT reported in strawberry leaves and consist of C–C-linked gallic acid monomers. Despite being the most abundant class of phenolics in strawberry leaves, the ET content showed a lower response to the variation in N treatments than the phenylpropanoids, which exhibited an increase in their content as the N supply decreased (Figure 5A and Supplementary Figures 6A,B). Weaker induction of ET biosynthesis could arise due to substrate competition for the synthesis of gallic acid and chorismic acid, the precursors for phenylpropanoids. Thus, regulation by N at the branch point of 3-dehydroshikimate is proposed to explain the variation in the abundance pattern of ETs and phenylpropanoids (Salminen and Maarit, 2011). Similar tradeoffs between gallic acid and phenylpropanoids have been reported in strawberry leaves (Kim et al., 2016). These observations highlight that the stress stimuli can be utilized to regulate carbon partitioning between the shikimate and the phenylpropanoid pathways, and N appears to be a potential candidate for imposing this modulation.

The second branch point of phenolic biosynthesis occurs at coumarate in the phenylpropanoid pathway, resulting in the biosynthesis of the derivatives of simple phenolic acids and flavonoids. Among simple phenolic acids, hydroxycinnamates conjugated with flavonols exhibit a higher free radical scavenging capacity than free hydroxycinnamates (Martinez et al., 2016). Thus, the similar ratio of hydroxycinnamates:flavonoids (Supplementary Figure 9) across the N treatments observed in the present study could represent the effective regulation of carbon partitioning for the biosynthesis of substrates, which in turn could be used to generate conjugated phenolics that are more reactive. The abundance of different flavonoid classes, including flavanones, flavones, flavonols, flavan-3-ols, and proanthocyanidins, was inversely related to N supply, which is in agreement with previous research across different species (Strissel et al., 2008; Becker et al., 2015). The reduced N treatment of 8 mM resulted in a maximum increase in the flavonol content over that of the flavones in both cultivars. Flavonols, because of their 3’-hydroxyl group and lower redox potential, are more potent free radical scavengers than flavones (Dwivedi et al., 2016).

The synthesis of flavan-3-ols and their oligomers (proanthocyanidins/condensed tannins) shares common steps in the flavonoid pathway with the synthesis of anthocyanins and flavonols. In the present study, both flavonol and flavan-3-ols increased with the decrease in N supply, and the anthocyanin abundance was negligible. However, the flavan-3-ols exhibited a much higher fold change with the decrease in N than the flavonols. Dihydroflavonols are utilized in the biosynthesis of not only flavonols but also leucoanthocyanidins, which are utilized for the biosynthesis of anthocyanins and flavan-3-ols. The anthocyanin content observed in our study was lower than the content previously reported under cold treatment in leaves and fruits (Koehler et al., 2015; Tanou et al., 2017). The reduction of anthocyanins to flavan-3-ols by anthocyanin reductases (ANRs) has previously been reported to contribute a major share of the flavan-3-ols in tobacco (Luo et al., 2016), cotton (Zhu et al., 2015), white clover (Abeynayake et al., 2012), grape (Bogs et al., 2007), and strawberry (Fischer et al., 2014). Thus, the observed lower abundance of anthocyanin and higher abundance of flavan-3-ols under N limitation could be attributed to upregulation of the ANR. A similar decrease in anthocyanin content and rechanneling of the metabolic flux to flavan-3-ols was reported in strawberry fruits when UDP-glucose:anthocyanidin glucosyl transferase (FaGT1) was downregulated (Griesser et al., 2008). However, more investigation is required to establish the relationship between N and the activity of the enzymes involved in biosynthesis.

### Variation in the Composition of Phenolic Classes in Response to Varied Nitrogen Supply

The simple galloyl glucoses, which are the first branch-off products from the shikimate pathway and the precursors of HT, were higher at lower N (control and 8 mM N) levels. Glucogallin constituted a major (>70%) galloyl glucose ester in the leaves of both strawberry cultivars across all N treatments. A decrease in glucogallin followed by an increase in digalloylglucose has been reported in mature birch leaves and is proposed to be a result of the efficient utilization of glucogallin (Salminen et al., 2001). The higher content of glucogallin in proportion with tri- and tetra-galloyl glucose across all N treatments in both cultivars could indicate reduced kinetics of the galloyltransferases compared to glucosyltransferase in the applied N treatment. Across plant species, it has been reported that GT and ET are not simultaneously produced (Salminen and Maarit, 2011) and that strawberry leaves have a higher abundance of ET compared to polymeric GTs (Kim et al., 2016; Kårlund et al., 2016; Michalska et al., 2017). The preferential biosynthesis of ET in strawberry leaves over GT could be due to preferential oxidation of the branch point substrate pentagalloylglucose, as opposed to the formation of a depside bond between the gallic acid subunits.

The simple phenolic acids from the study were categorized as hydroxybenzoates and hydroxycinnamates. The presence of a *p*-hydroxy group and an additional carbon side chain structure on the phenol ring has been shown to increase radical scavenging activity (Natella et al., 1999). The observed higher content of hydroxycinnamates (coumaric and ferulic acids) compared to the hydroxybenzoates (protocatechuic acid) under N limitation could represent a structure-dependent preferential accumulation of more effective antioxidants. In addition, the preferential utilization of hydroxycinnamates in conjugation to flavonols could explain the increased biosynthesis of hydroxycinnamates over hydroxybenzoates.

Further along the flavonoid pathway, the flavone apigenin along with the flavonols quercetin and kaempferol (*via* dihydroflavonols) constituted the most abundant compounds derived from the two branches of the flavanone naringenin in both cultivars across all N treatments. Apigenin is often reported as the only flavone in strawberry fruits (Akhatou et al., 2017) and has been shown to have minimal antioxidant capacity compared to kaempferol and quercetin (Pannala et al., 2001; Sroka et al., 2015). In the present study, the decrease in the kaempferol:quercetin ratio with decreases in the N treatment was attributed to an increase in quercetin and not to a decrease in kaempferol. Regardless of the stress factor, quercetin is reported as a more abundant flavonol in strawberry fruits and leaves compared to kaempferol (Kårlund et al., 2016; Michalska et al., 2017). The dihydroxylated flavonol quercetin has higher superoxide radical scavenging activity than the monohydroxylated kaempferol (Marković et al., 2014; Zhang et al., 2015). The variation in the relative proportions of kaempferol and quercetin could be due to their differential stability under different N conditions. Previous studies in *Arabidopsis* have reported that glycosides of kaempferol are more stable than quercetin following 2 days of N starvation and high temperatures (Olsen et al., 2009). In accordance with previously reported studies, hexoses, pentoses, and glucuronides were the key glycosylated forms in the strawberry leaves (Supplementary Table 1; Hanhineva et al., 2008; Kim et al., 2016). Glycosylation and acylation not only improve the biological activity of flavonoids but also modify the physical parameters of solubility, thermal/light stability, and membrane permeability (Ogo et al., 2015). Furthermore, differences in the bioactivity of these flavonoids associated with the decorative modification on the flavan structure is well documented (Alseekh et al., 2020), the exact role of individual metabolites under specific stress cue needs more detailed evaluation.

In our study, catechin monomers and proanthocyanidin dimers had higher abundances under N limitation than the trimers and tetramers. The enzymatic (*via* polyphenol oxidases) and non-enzymatic (chemical oxidation) mechanisms of proanthocyanidin polymerization from monomers are widely debated (Xie and Dixon, 2005), making it difficult to ascertain the effect of N fertilization. A positive correlation has been demonstrated between the mean degree of polymerization and the antioxidant capacity (Zhou et al., 2014). Thus, despite the higher abundances of dimers across the treatments in both cultivars, the higher percentage increase in trimers with the reduction in N supply could be construed as the production of compounds with increased antioxidant capacity. The increase in radical scavenging activity with the increase in the polymerization from *n* = 1 to 4 and the reversal of that trend at higher degrees of polymerization was attributed to the steric hindrances contributing to the accessibility of hydroxyl groups (de Gaulejac et al., 1999). These findings support the close association of structural conformation with biological activity and require further studies to determine the relationship between N limitation and proanthocyanidins at a higher degree of polymerization.

### Variation in the Localization of Phenolic Classes in Response to Varied Nitrogen Supply

In the present study, acid hydrolysis of the residue left after the extraction of the soluble phenolics was performed for the determination of the bound form of the phenolic classes. Although the extractable form of the different phenolic classes increased with a decrease in N supply, the bound form of phenolics was similar across the different N treatments in both cultivars. A similar increase in soluble phenolics with an increase in N and relative stability in bound phenolics was reported in wheat (Stumpf et al., 2015). It has been proposed that genetic determinants primarily regulate the bound phenolics, and the soluble phenolics are primarily regulated by environmental cues (Fernandez-Orozco et al., 2010). Although the exact sequestration mechanism of phenolics onto the cell wall is currently unknown, it is proposed that the phenolic precursors are mobilized from the site of biosynthesis by vesicle trafficking or transporter proteins and polymerized on the structural components of the cell wall usually through peroxidase activity (Zhao et al., 2010). These cell wall-bound phenolics, characterized mainly by phenolic acids, act as a barrier for effectively absorbing harmful UV radiation and limiting water loss by maintaining the integrity and hydrophobicity of the cell wall (Hura et al., 2017). In accordance with the above theory, in the present study, there is a higher proportion of the simple phenolic acids that are bound to the cell wall than flavonols. In addition, an increase in porosity in the cell wall is proposed to facilitate tannin adsorption to the cell walls and decrease tannin extractability (Bindon et al., 2014). Thus, the observed higher proportion of phenolics in the soluble form could be because of the increased porosity and reduced stability of the cell membrane under N limitation.

## Conclusion

The structural diversity within the phenolics differentially regulates their biological functions. Our results show that, along with the variation in the quantity of different phenolic classes, N supply influences the compositional variation both within and across different phenolic classes and their association with cellular components. Overall, the phytochemical content increased with decreasing N availability. However, the proportional increase in the quantity of proanthocyanidins compared to the other phenolic classes highlights the non-linear response of phytochemicals to the applied N treatments. The increased preferential accumulation of metabolites with selective structural modifications with enhanced antioxidant capacity, such as hydroxylation, glycosylation, and ring conjugation, under a low N supply, suggests a potential adaptation of these plants to abiotic stress. The specific influence of N supply on the transportable phenolics with higher antioxidant capacity, but not on the bound phenolics, suggests the potential for precise management of moderate nutrient stress as a means to increase the phytochemical content and nutritive value of food crops.

## Data Availability Statement

The original contributions presented in the study are included in the article/Supplementary Material, further inquiries can be directed to the corresponding author/s.

## Author Contributions

NT designed the experiment. AN conducted the experiment and analyzed the data. Both authors contributed to the manuscript preparation.

## Conflict of Interest

The authors declare that the research was conducted in the absence of any commercial or financial relationships that could be construed as a potential conflict of interest.
