# Supplementary material for: Nitrogen Fertilization Influences the Quantity, Composition, and Tissue Association of Foliar Phenolics in Strawberries
Source: Front Plant Sci. 2021 Apr 20;12:613839. doi: 10.3389/fpls.2021.613839 (PMC8093403; doi:10.3389/fpls.2021.613839)
Supplement: Supplementary file 1 [file Data_Sheet_1.docx]

Nitrogen fertilization influences the quantity, composition, and tissue-association of foliar phenolics in strawberries.

A

**Authors:** Ashwini Sushil Narvekar, Nishanth Tharayil


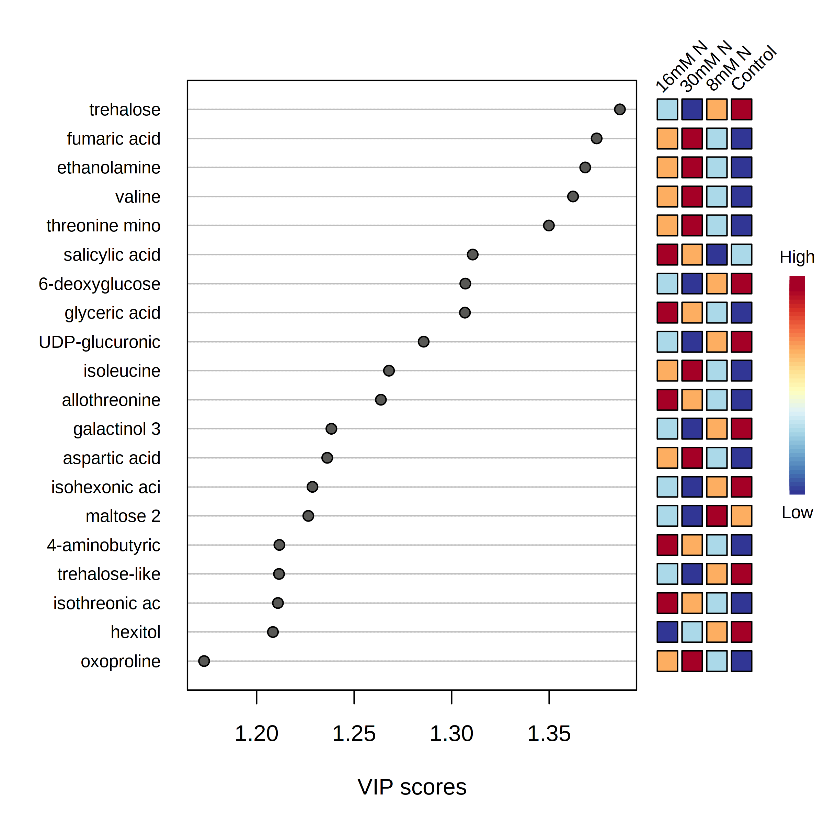

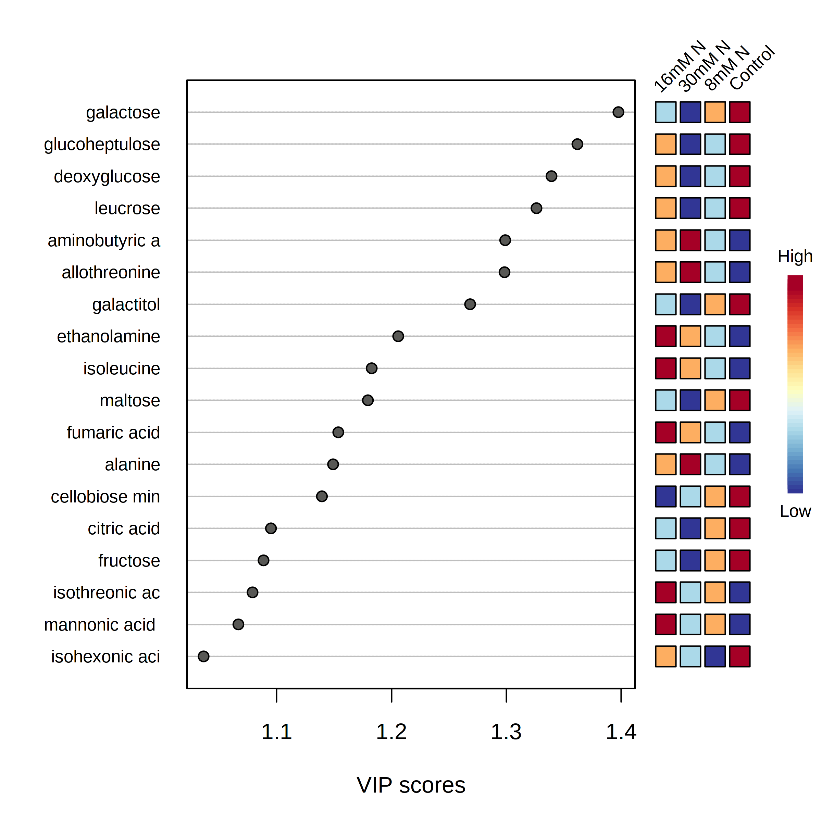
**Affiliation**: Plant and Environmental Sciences, Clemson University, Clemson SC.

B

Supplementary Figure 1: Variable importance in projection (VIP) plot in *cv*. Albion (A) and Camarosa (B) displays primary metabolite features of VIP >1, identified by PLS-DA. Colored boxes on right indicate relative concentration of corresponding metabolite the different N treatment.


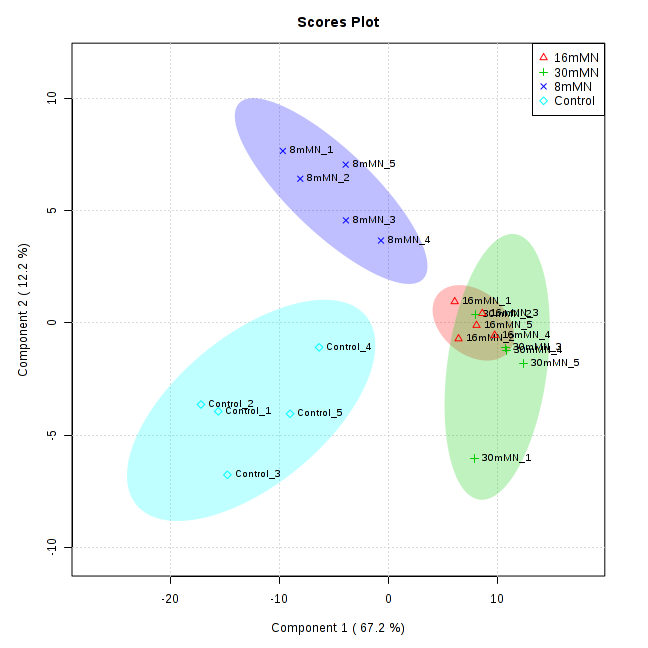

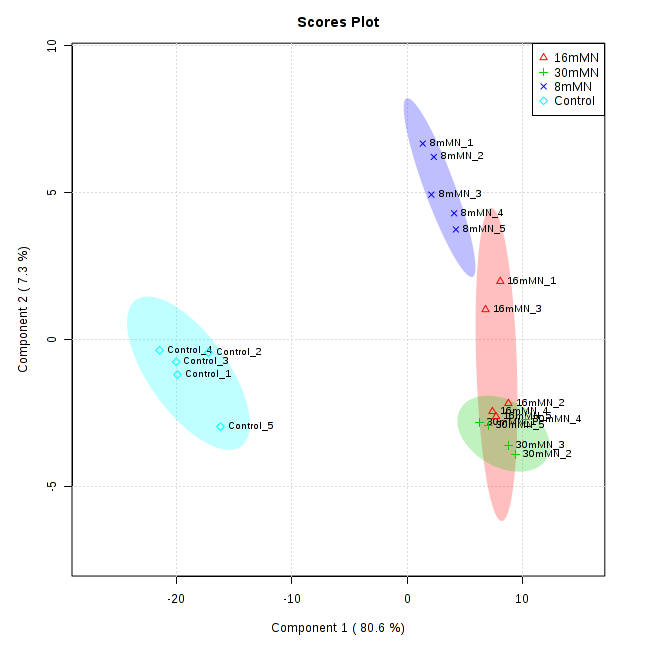


A

B

Supplementary Figure.2. Partial least squares Discriminant Analysis (PLS-DA) score plot of first two principal components of the analysis of 149 secondary metabolites of strawberry cv. Albion (A) and cv. Camarosa (B) exposed to four levels of nitrogen (Green: 30mM N, Red: 16mM N, Blue: 8mM N and Turquoise: Control). The permutation cross-validation of the PLS-DA model had P = 0.0005 over 2000 iterations. The ellipse represents a 95% confidence interval.


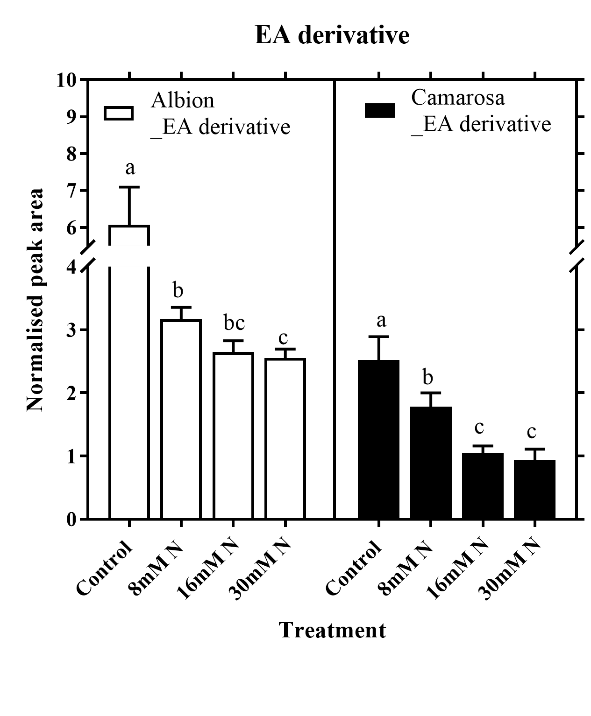


B


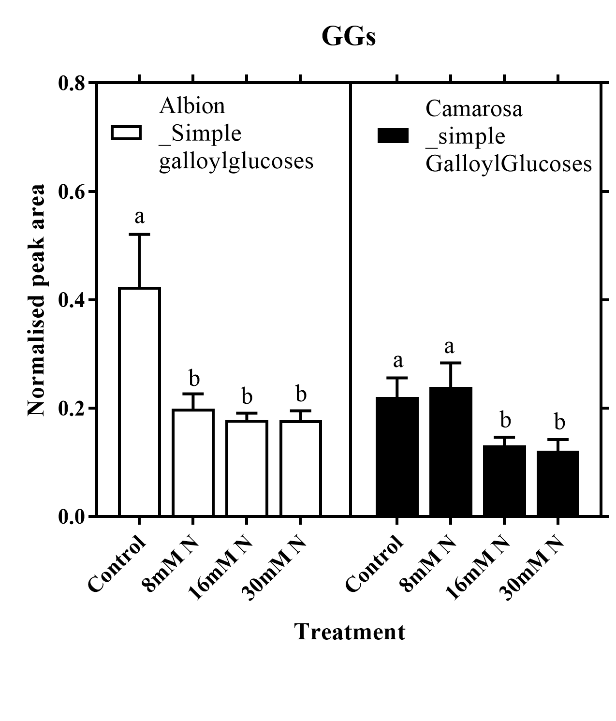


A

Supplementary Figure.3.:The bar graph represent mean ± SD of peak area normalized with C^13^ resveratrol of simple galloylglucoses (A) and ellagic acid derivatives (B) in the respective N treatments of *cv*. Albion (open bar) and *cv*. Camarosa (closed bar). Bars with the same letters are not significantly different at the 0.05 confidence level (Tukey's HSD).


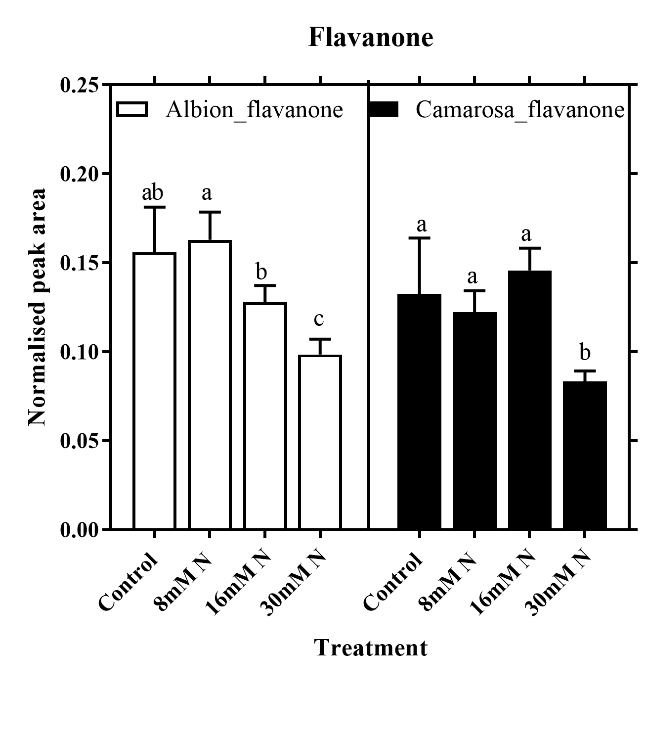

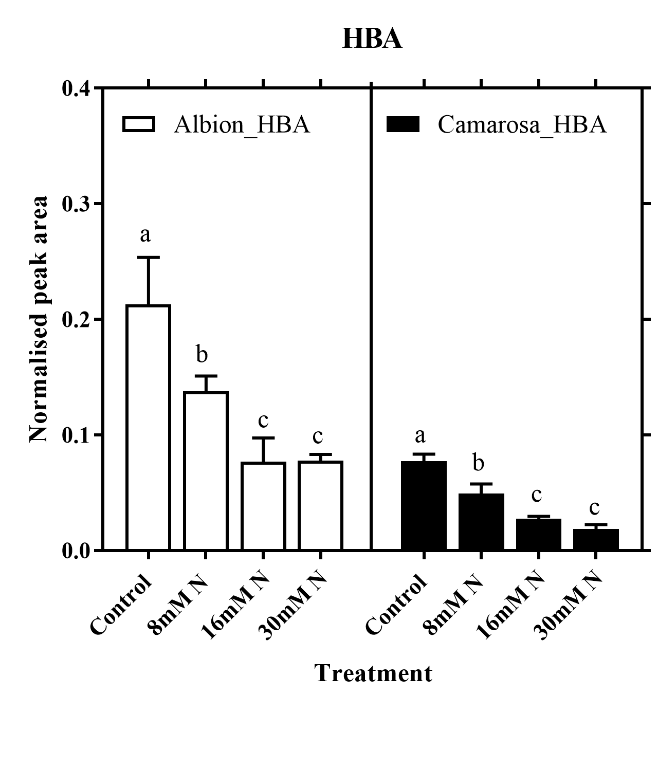

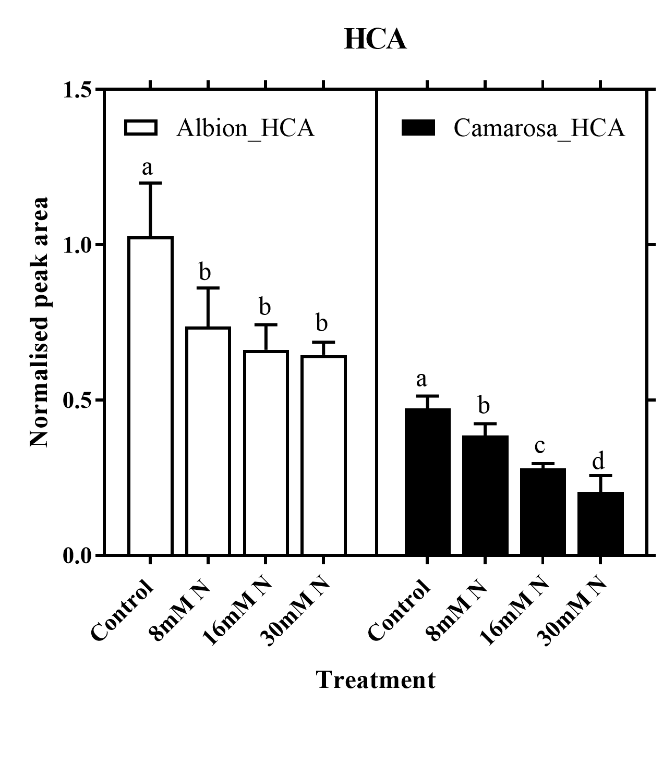

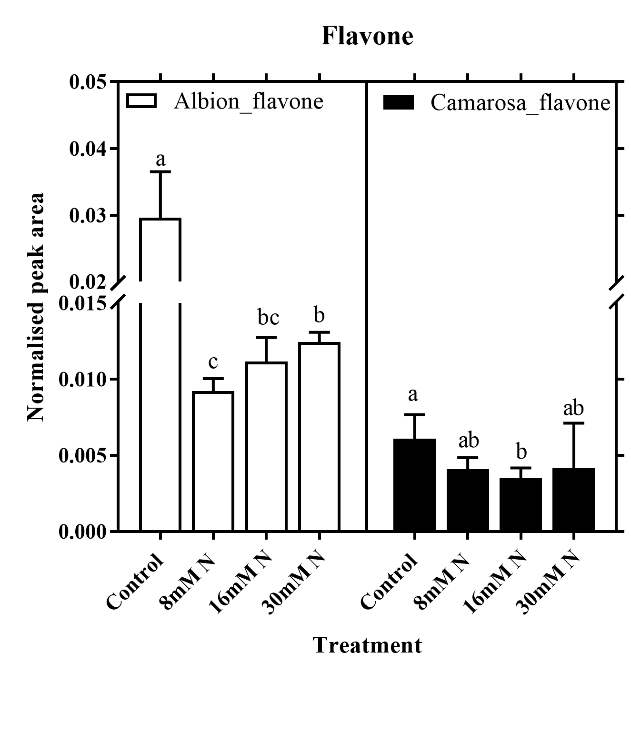


C

D

A

B

Supplementary Figure 4 : The bar graph represent mean ± SD of peak area normalized with C^13^ resveratrol of hydroxybenzoates (A), hydroxycinnamates (B), flavanones (C) and flavones (D) in the respective N treatments of *cv*. Albion (open bar) and *cv*. Camarosa (closed bar). Bars with the same letters are not significantly different at the 0.05 confidence level (Tukey's HSD).


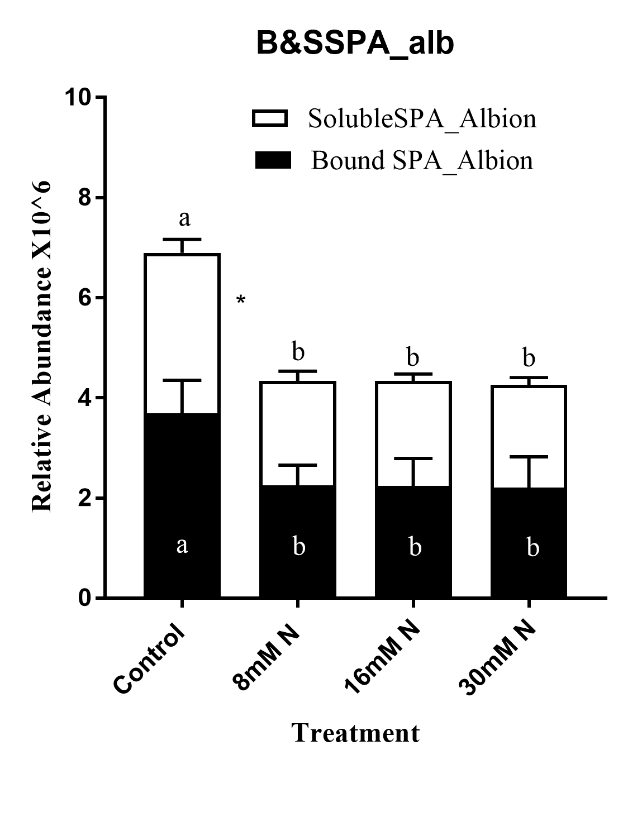

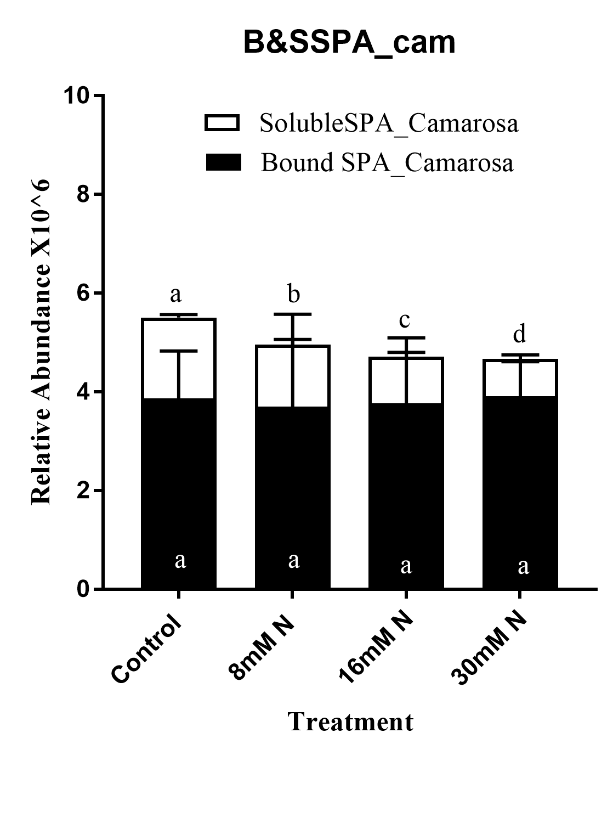


A

B


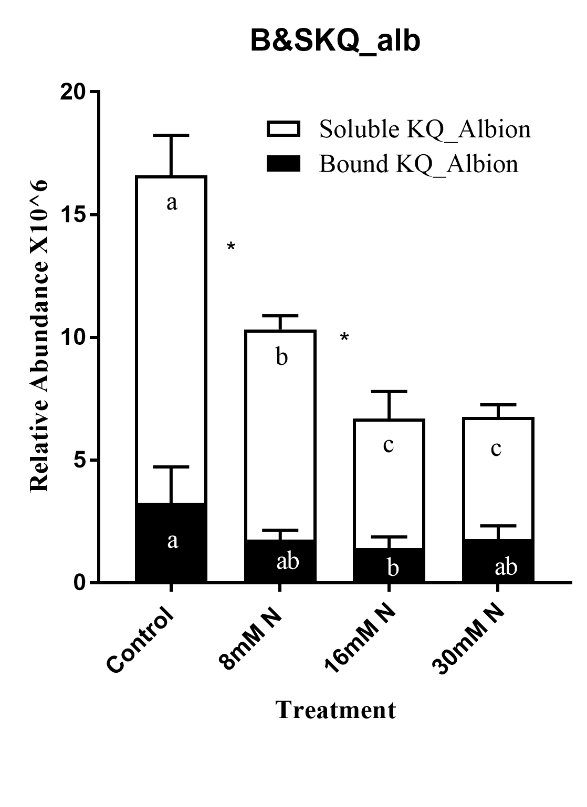

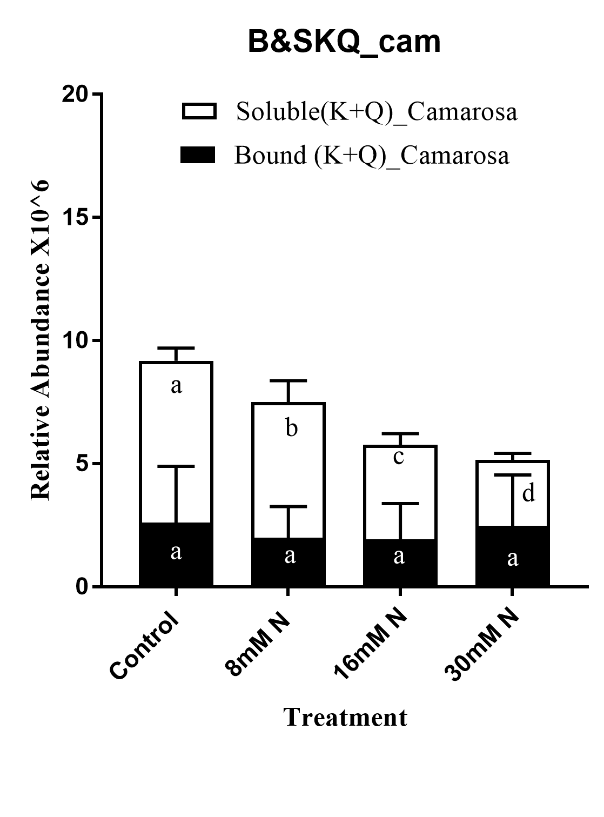


C

D

Supplementary Figure .5: The stacked bar graph represent mean ± SD of relative abundances of simple phenolic acids (A and B) and flavonol (Kaempferol and Quercitin aglycone (C and D)) for the respective N treatments in *cv*. Albion (A and C) and *cv*. Camarosa (B and D). The legends show the corresponding color and groups. For each compartment, error bars with the same letters are not significantly different at 0.05 significance. The stacks within a bar is not compared due to the potential difference in ionization of these compounds. The asterisk mark represents a significant difference between total content.


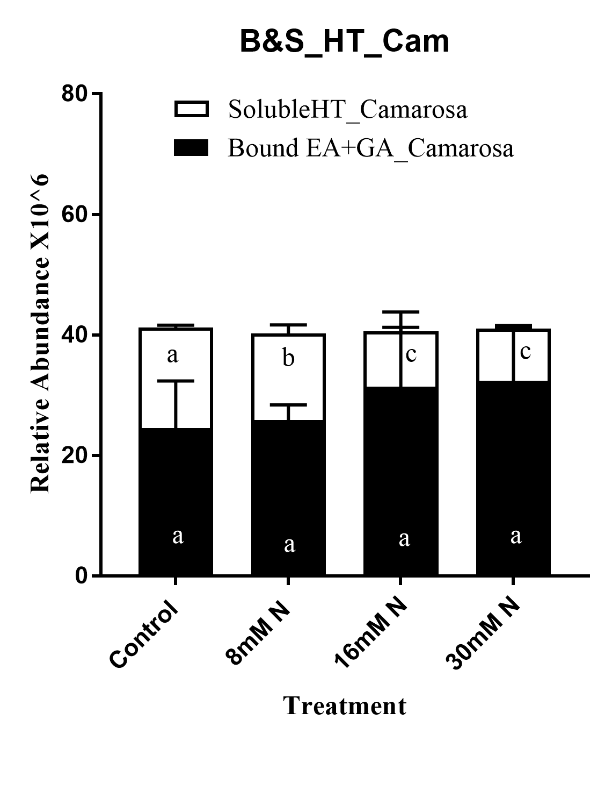

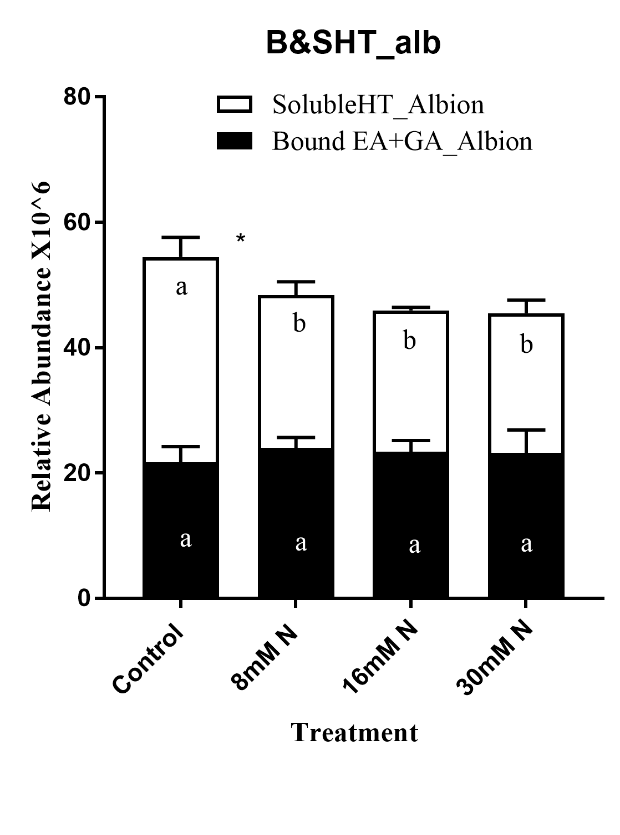


A

B


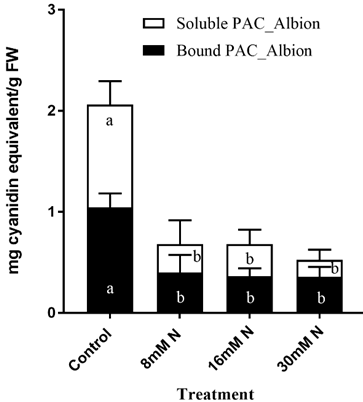

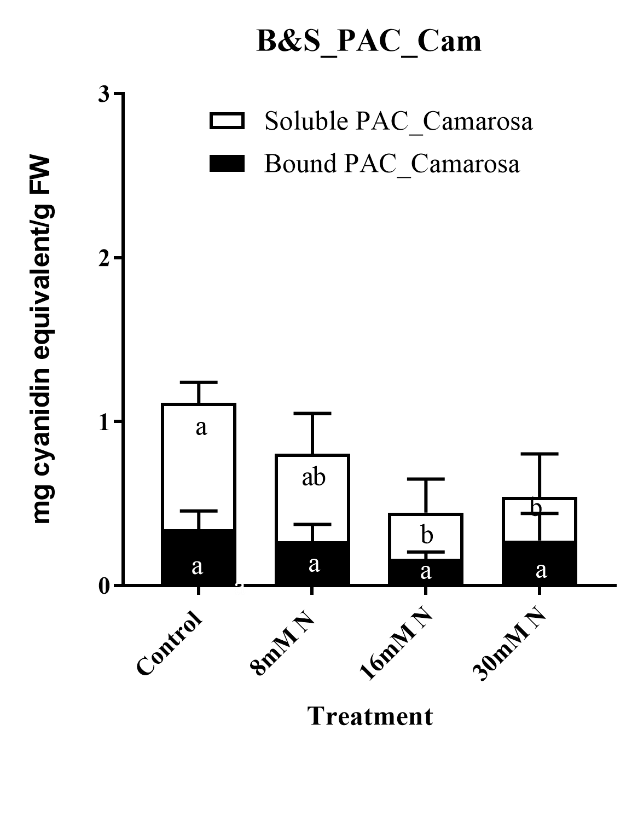


D

C

Supplementary Figure.6: The stacked bar graph represent mean ± SD of relative abundances of total hydrolysable tannins and its precursor monomers from the acid hydrolysis of the residue (A and B). The stacked bar graph C and D represent mean ± SD of mg of cyanidin equivalent/g FW for the respective N treatments in *cv*. Albion (C) and *cv*. Camarosa (D). The legends show the corresponding color and groups. For each compartment, error bars with the same letters are not significantly different at 0.05 significance level. There is no comparison between the stacks within a bar.


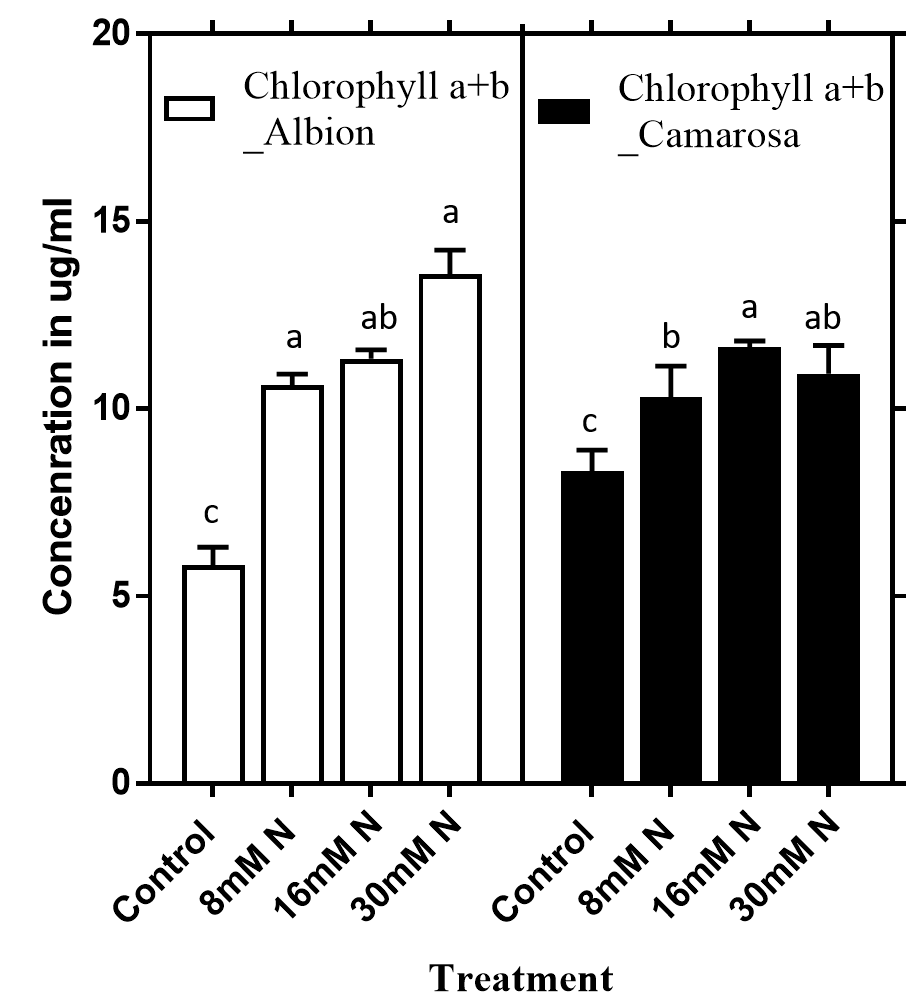

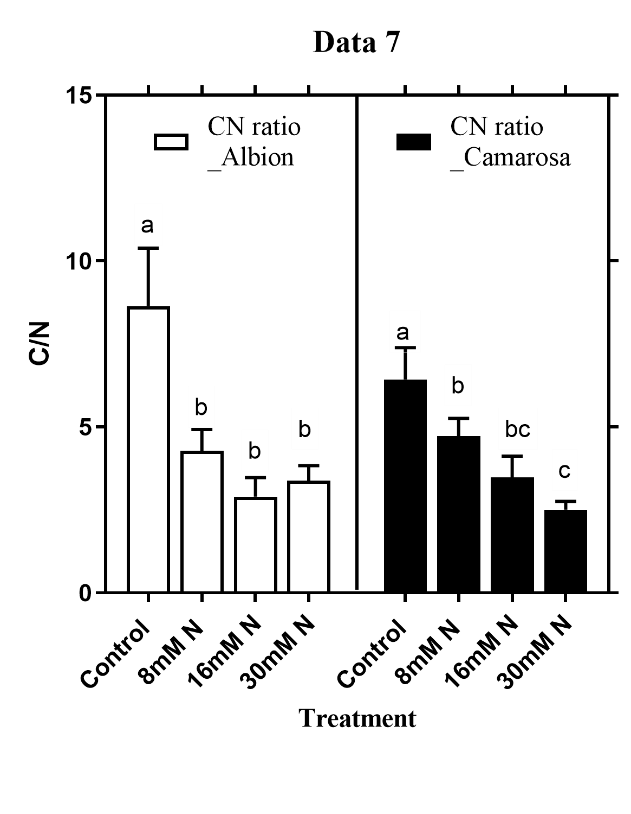


Supplementary Figure. 7: The bar graph represent mean ± SD chlorophyll content (A) and the carbon: nitrogen ratio (B) in the respective N treatments. The open bars and closed bar represent data from *cv*. Albion and from cv. Camarosa respectively.


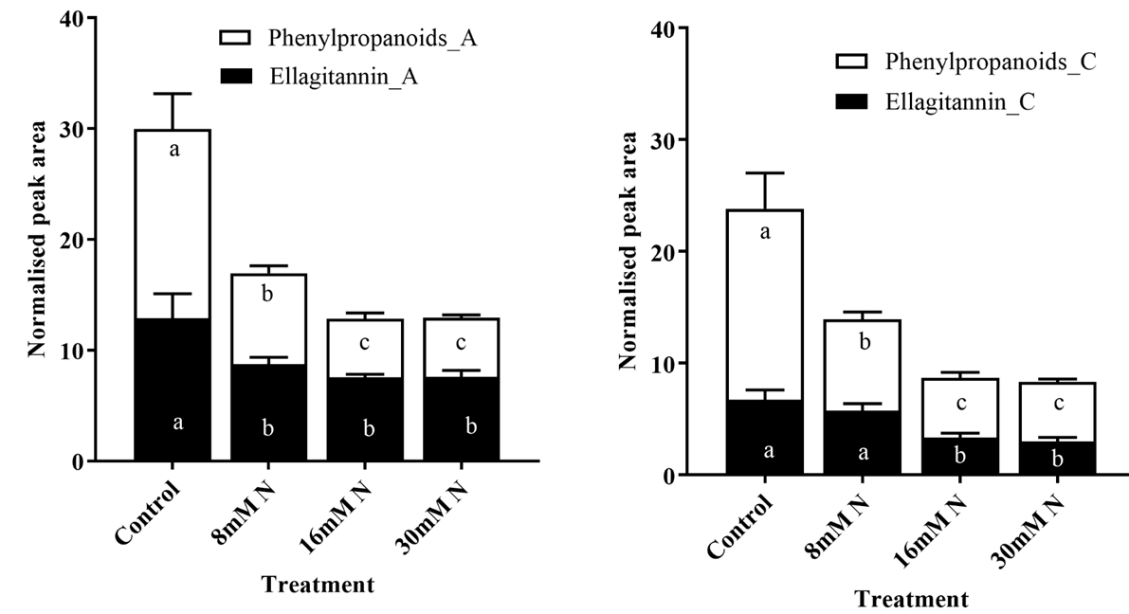


Supplementary Figure 8: The stacked bar graph represent mean ± SD of peak area normalized with C^13^ resveratrol of total ellagitannin (closed bars) and phenylpropanoids (open bars) of the respective N treatments in *cv*. Albion (C) and *cv*. Camarosa (D). The legends shows the corresponding color and subgroups. Bars with the same letters are not significantly different at 0.05 confidence level (Tukey's HSD). There is no comparison between the stacks within a bar.

.


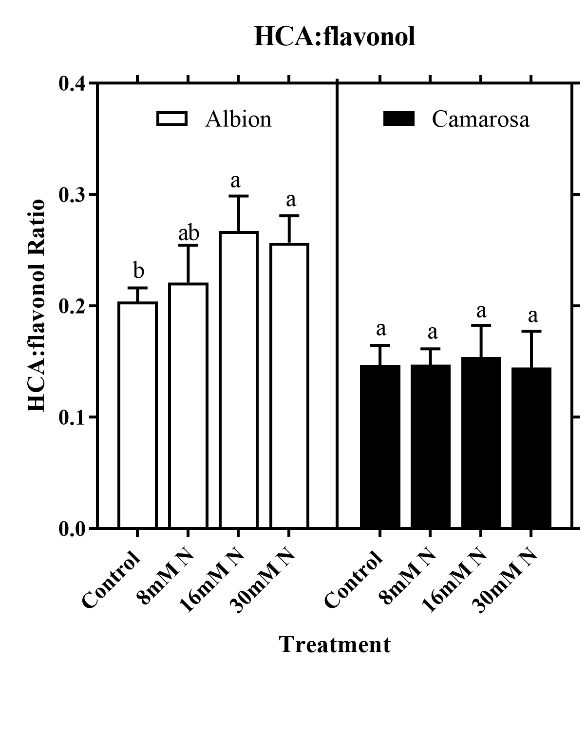


Supplementary Figure 9: The bar graph represents hydroxycinnamate:flavonol ratio mean ± SD of peak area normalized with C^13^ resveratrol in the respective N treatments. The open bars and closed bar represent data from *cv*. Albion and from *cv*. Camarosa respectively.


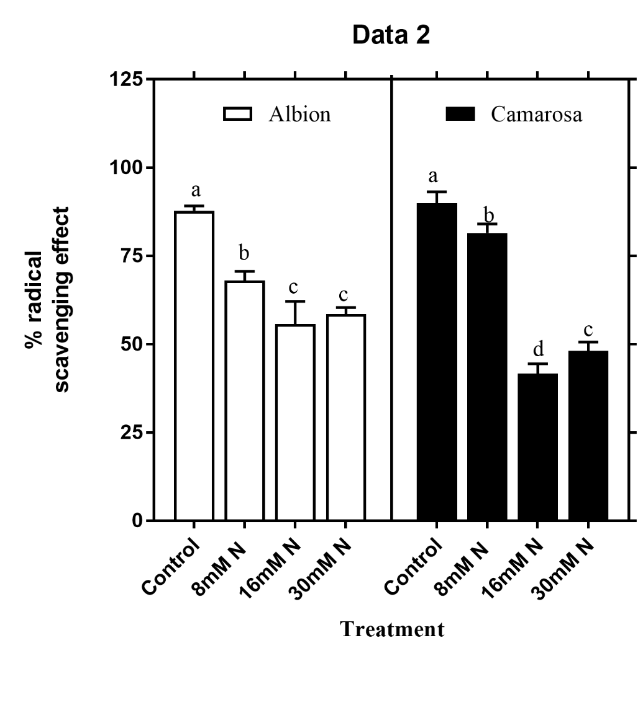


Supplementary Figure 10: The b represent mean ± SD of % scavenging of DPPH radical in the respective N treatments in *cv*. Albion (open bars) and *cv*. Camarosa (closed bars). Bars with the same letters are not significantly different at 0.05 confidence level (Tukey's HSD).
